# Supplementary material for: Concomitant ablation of SOS1 and SOS2 triggers a lethal phenotype involving compromised intestinal integrity and widespread septicemia
Source: Cell Death Dis. 2026 Aug 12;17(1):949. doi: 10.1038/s41419-026-09185-z (PMC13745597; doi:10.1038/s41419-026-09185-z)
Supplement: Supplementary file 1 [file 41419_2026_9185_MOESM1_ESM.pdf]

Concomitant ablation of SOS1 and SOS2 triggers a lethal phenotype involving compromised intestinal integrity and widespread septicemia

Andrea Olarte-San Juan, Pablo Rodríguez-Ramos, Alba Díaz-Aguilera, Nuria Calzada, Carmela Gómez, Rocío Fuentes-Mateos, Alberto Fernández-Medarde, Rubén Nogueiras, David Díaz, Eugenio Santos, and Rósula García-Navas.

Supplemental material

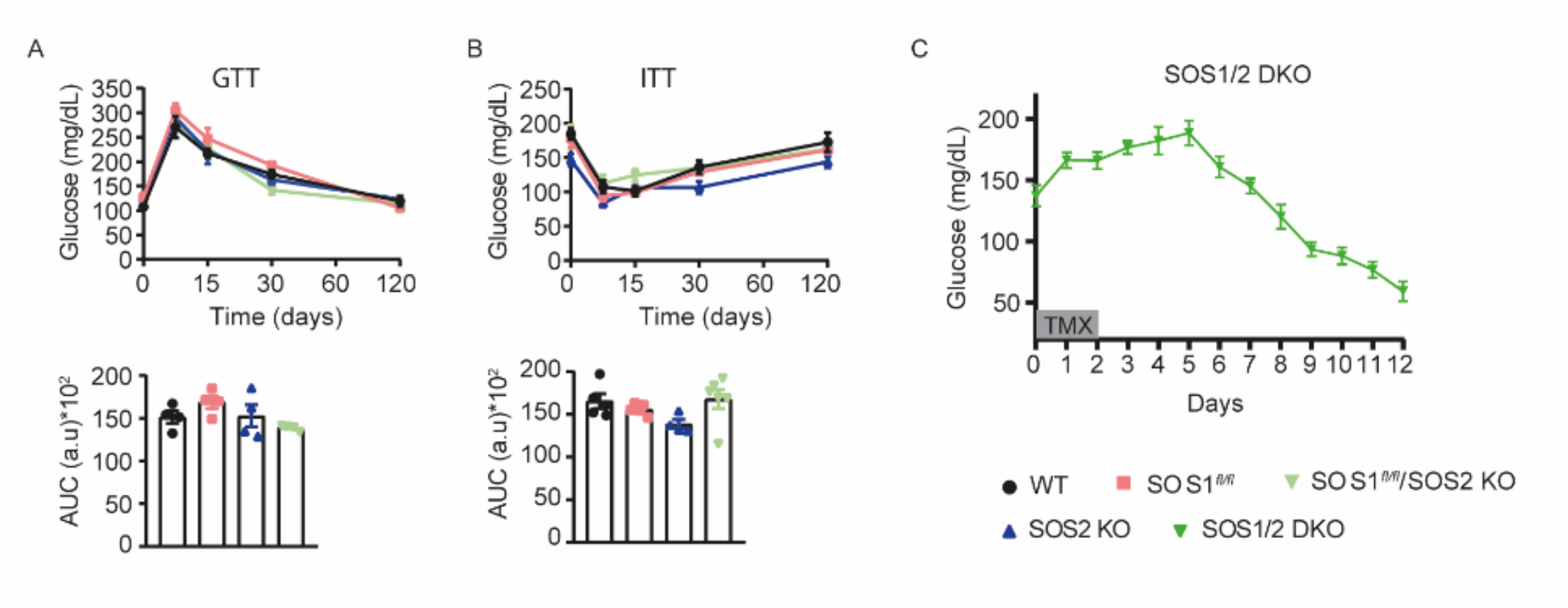

Figure S1: Glucose and insulin tolerance tests reveal no genotype-dependent differences before TMX treatment, whereas SOS1/2 DKO mice exhibit reduced glucose levels and less severe colonic injury following TMX administration.

(A) A glucose tolerance test (GTT) was performed before TMX treatment in the indicated genotypes. The upper panel shows blood glucose levels over time, and the lower panel shows quantification of the area under the curve (AUC).

(B) An insulin tolerance test (ITT) was performed before TMX treatment in the indicated genotypes. The upper panel shows blood glucose levels over time, and the lower panel shows quantification of the area under the curve (AUC).

(C) Serum glucose was measured during TMX treatment on SOS1/2 DKO mice.

(D) Representative hematoxylin and eosin (H&E)- stained sections of the large intestine from all experimental groups on day 12 after TMX treatment, and large intestine length quantification from each experimental group on day 12 after TMX treatment.

Data expressed as the mean ± s.e.m. for at least n=4 for each genotype per day. Statistical analysis was performed using one-way ANOVA, with Tukey's post hoc test. Significance indicators: * vs. WT, & vs. SOS1 KO and # vs. SOS2 KO; **, &&, ## p<0.01.

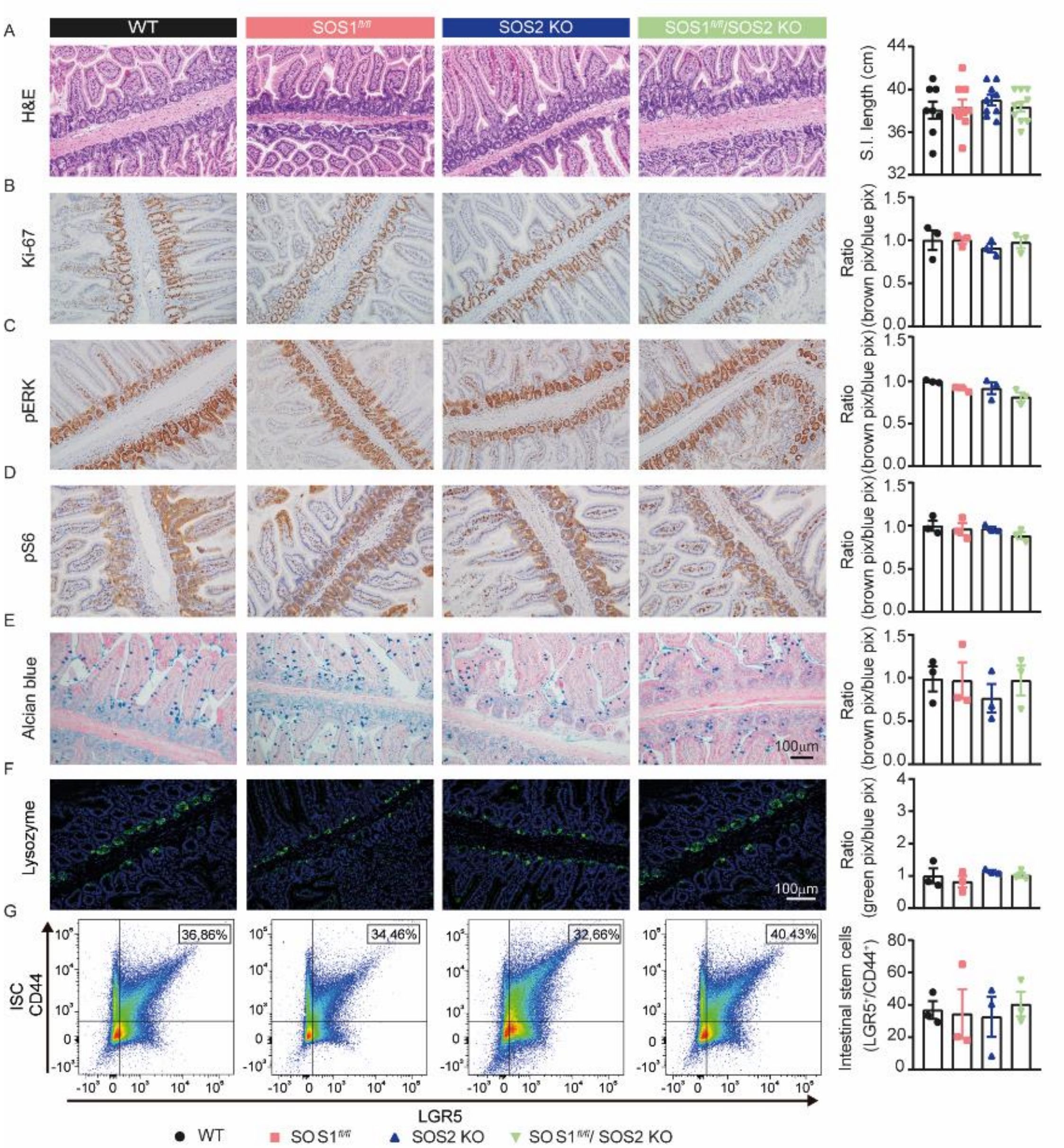

Figure S2: Characterization of the small intestine reveals no genotype-dependent differences before TMX treatment.

(A) Representative hematoxylin and eosin (H&E)- stained sections of the small intestine from all experimental groups before TMX treatment, and small intestine length quantification from each experimental group before TMX treatment.

(B-D) Representative immunohistochemistry images showing Ki-67 (B), pERK (C), and pS6 (D) staining in WT, SOS1 KO, SOS2 KO, and SOS1/2 DKO mice before TMX treatment, with quantification.

(E) Representative alcian blue images of the small intestine of the four genotypes under study before TMX treatment, with quantification.

(F) Representative lysozyme immunofluorescence images of the small intestine of the four genotypes under study before TMX treatment, with quantification.

(G) Representative FACS profiles of $LGR5^+/CD44^+$ expression and the frequency of $LGR5^+/CD44^+$ Intestinal Stem Cells (ISC) quantification from each experimental group before TMX treatment.

Data expressed as the mean ± s.e.m. for at least n=4 for each genotype.

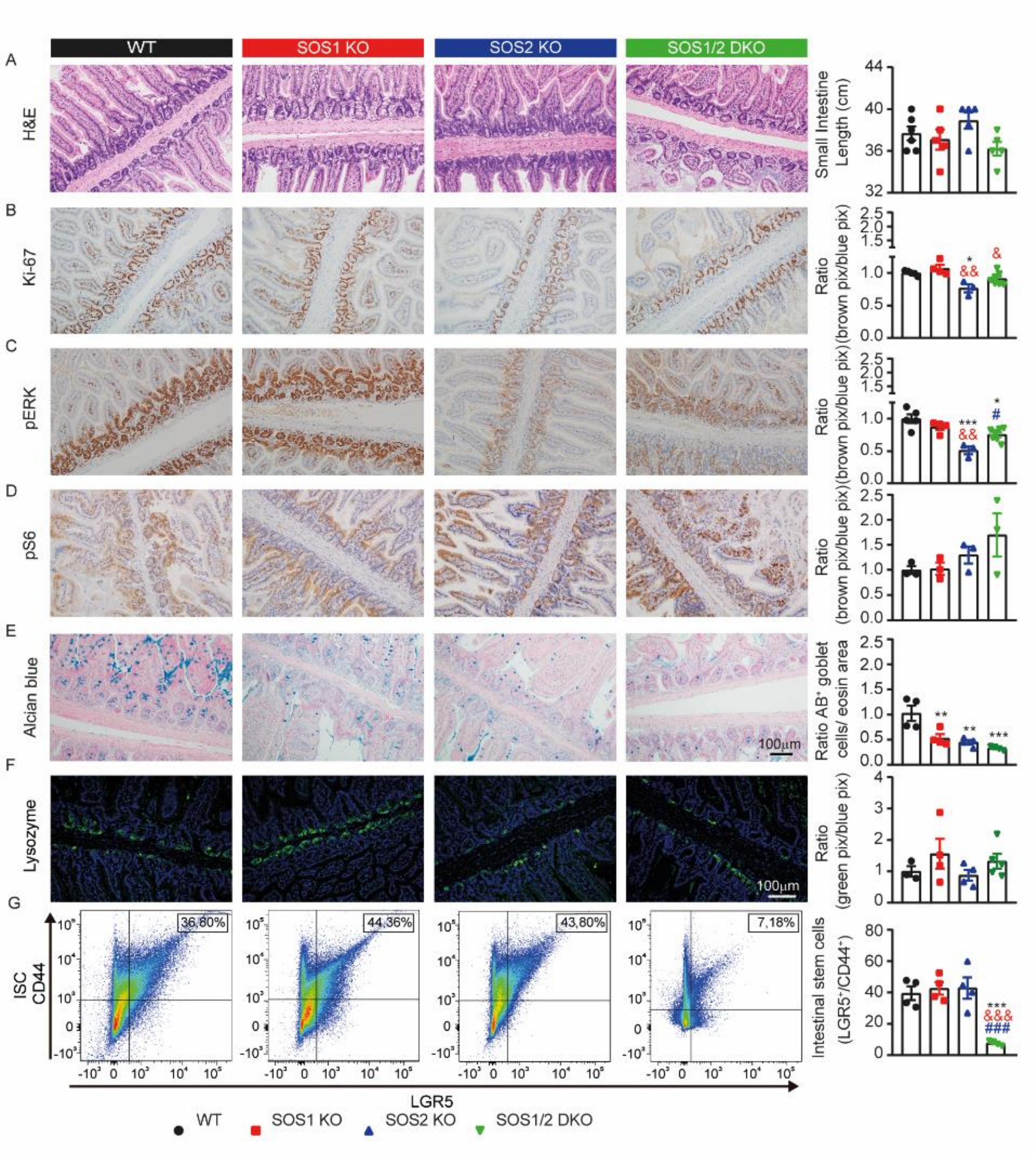

Figure S3: Ki-67 staining and ERK phosphorylation were reduced in SOS2 KO and SOS1/2 DKO mice after 6 days of TMX treatment.

(A) Representative hematoxylin and eosin (H&E)- stained sections of the small intestine from all experimental groups on day 6 after TMX treatment, and small intestine length quantification from each experimental group on day 6 after TMX.

(B-D) Representative immunohistochemistry images showing Ki-67 (B), pERK (C), and pS6 (D) staining in WT, SOS1 KO, SOS2 KO, and SOS1/2 DKO mice 6 days after TMX treatment, with quantification.

(E) Representative alcian blue images of the small intestine of the four genotypes under study on day 6 after TMX treatment, with quantification.

(F) Representative lysozyme immunofluorescence images of the small intestine of the four genotypes under study on day 6 after TMX treatment, with quantification.

(G) Representative FACS proles of LGR5$^+$/CD44$^+$ expression and the frequency of LGR5$^+$/CD44$^+$ Intestinal Stem Cells (ISC) quantification from each experimental group on day 6 after TMX treatment.

Data expressed as the mean ± s.e.m. for at least n=4 for each genotype. Statistical analysis was performed using one-way ANOVA, with Tukey's post hoc test. Significance indicators: * vs. WT, & vs. SOS1 KO and # vs. SOS2 KO; *,& $p<0.05$; **, && $p<0.01$; *** $p<0.001$.

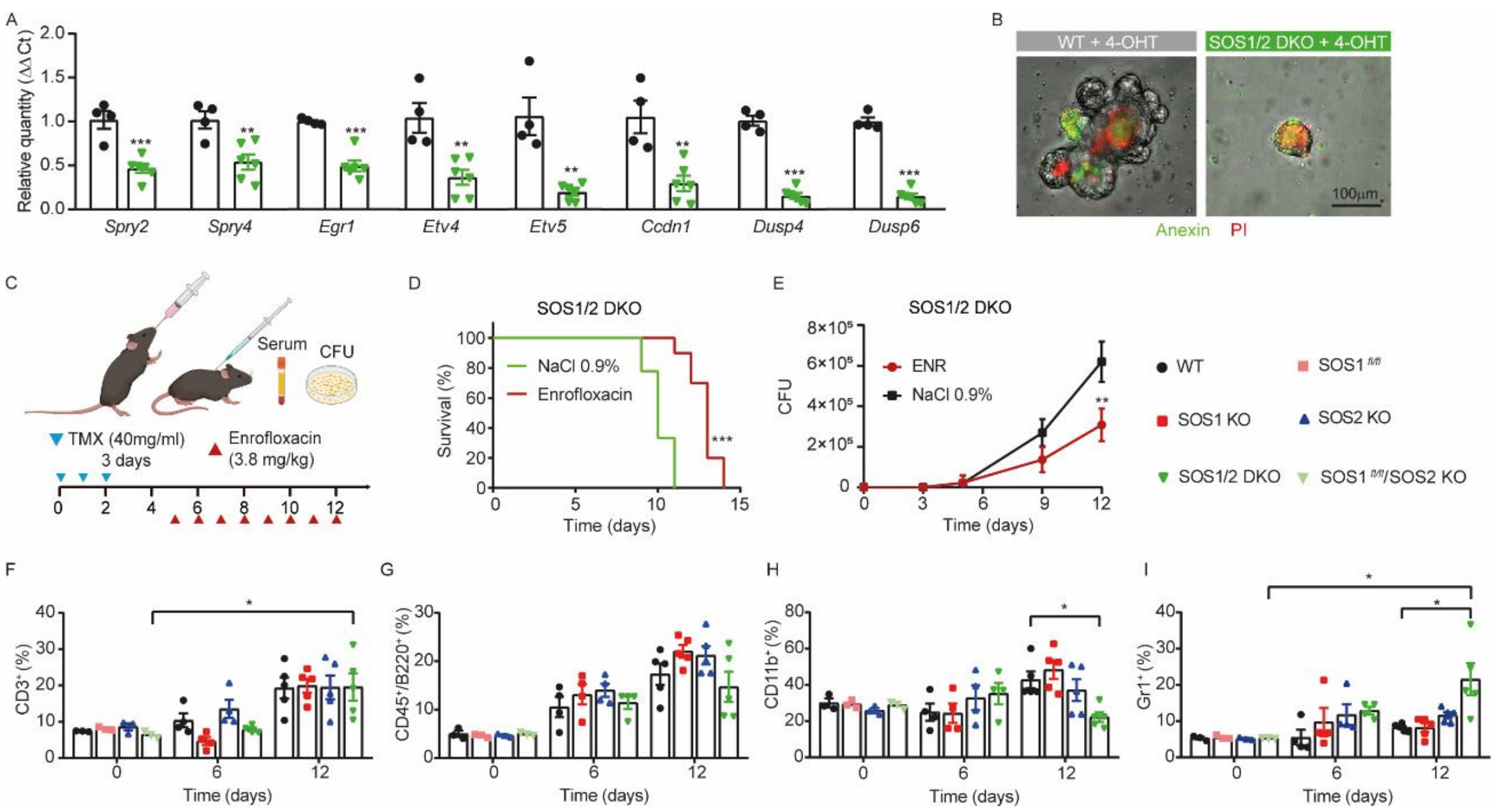

Figure S4: SOS1/2 deficiency drives intestinal epithelial collapse, ERK signaling impairment, and systemic immune dysregulation.

(A) mRNA expression levels of the indicated genes in crypt-enriched intestinal epithelial cells isolated from the small intestine of WT, SOS1 KO, SOS2 KO, and SOS1/2 DKO mice on day 12.

(B) Representative brightfield/fluorescence overlay images of small intestinal organoids from WT and SOS1/2 DKO mice cultured in the presence of 4-OHT. Annexin V-FITC (green, early apoptosis) and propidium iodide (PI, red, cell death) staining are shown.

(C) Schematic representation of the experimental setup of the antibiotic treatment experiment. SOS1/2 DKO mice received either NaCl 0.9% or enrofloxacin (3.8 mg/kg, subcutaneously) daily, starting after TMX induction. *Created in BioRender. https://BioRender.com/dornmic.*

(D) Kaplan-Meier survival curves of SOS1/2 DKO mice treated with enrofloxacin (ENR) or vehicle (0.9% NaCl).

(E) Quantification of CFUs in blood from SOS1/2 DKO mice treated with ENR or NaCl over time.

(F-I) The percentages of $CD3^+$, $CD45/B220^+$, $CD11b^+$, and $Gr1^+$ cells in peritoneal lavage were assessed on days 0, 6, and 12 of TMX treatment across all genotypes.

Data expressed as the mean ± s.e.m. of at least 30 organoids from a minimum of 3 mice per genotype. For *in vivo* experiments, n ≥ 4 mice per genotype. Statistical analysis was performed using one-way ANOVA, with Tukey's post hoc test, and comparison of survival curves using the Log-rank (Mantel-Cox) test. Significance indicators: * vs. WT; * p<0.05, **p<0.01; *** p<0.001.

Table S1: Primer sequences used for qRT-PCR.

Gene	Forward	Reverse
mB2M	ATGGGAAGCCGAACATACTG	CAGTCTCAGTGGGGGTGAAT
Lyz	GGGAACCTGTGACCTGTCTT	GCCTCATGACACTGGGAACA
Defa5	TTGTCCTCCTCTCTGCCCTT	GACACAGCCTGGTCCTCTTC
Axin2	AGCTTTGAGCCTTCAGCATC	GCTCCAGAAGATCACAAAGAGC
Sox9	TCCAAACAGGCAGGGAGATTC	GCCAGATGGACCCACCAGTAT
cMyc	GATGAAATAGGGCTGTACGGAG	GCTGTTTGAAGGCTGGATTTC
Etv4	CGACTCAGATGTCCCTGGAT	GCCTGTCCAAGCAATGAAAT
Etv5	GGGAAATCTCGATCAGAGG	GGAGCATGAAGCACCAAGTT
Ccnd1	TGCGTGCAGAAGGAGATTGT	CTCACAGACCTCCAGCATC
Spry4	TGGAGCGATGCTTGTGACT	GAGGCTGGAGGTCCTGAAC
Spry2	CAGAGTTTGGAAAGAAGGA	CAGGTCTTGGCAGTGTGTT
Dusp6	ATGTGTGACGACTCGTACA	GCGTCGGAAATGGCGATCT
Dusp4	CTCATAGCCACCTTTAAGC	CGTGCGCTGCAATACCATC
Tjp1	TGGAATTGCAATCTCTGGTG	CTGGCCCTCCTTTTAACACA
Tjp2	ATGGGAGCAGTACACCGTGA	TGACCACCCTGTCATTTTCTTG
Ocln	GCTGTGATGTGTGTGAGCTG	GACGGTCTACCTGGAGGAAC
Cldn2	GCTCCGTGAGTATCTGGTCG	GTCTCTGGCAAGCTGACTTCT
Cldn3	AAGCCGAATGGACAAAGAA	CTGGCAAGTAGCTGCAGTG
Cldn4	CGCTACTCTTGCCATTACG	ACTCAGCACACCATGACTTG

Table S2: Progressive changes in haematological parameters following experimental induction.

Immune profiling of WT, SOS1 KO, SOS2 KO, and SOS1/2 DKO at days 0, 6, and 12 during TMX treatment.

Data expressed as the mean ± S.D. for at least n=3 for each genotype per day. Statistical analysis was performed using one-way ANOVA, with Tukey's post hoc test. Significance indicators: * vs. WT, & vs. SOS1 KO, and # vs. SOS2 KO; *, &, # p<0.05; ## p<0.01; ***, &&&, ### p<0.001.

		WT				SOS1				SOS2KO				SOS1/2 DKO			
Day	Param.	Mean	SD	N	Stats	Mean	SD	N	Stats	Mean	SD	N	Stats	Mean	SD	N	Stats
0	WBC	8,56	2,20	5		8,60	1,18	5		8,78	2,14	5		9,51	2,00	3	
6		4,71	2,47	3		2,43	0,40	6	##	6,05	2,15	5		8,23	0,70	3	* &&&
12		3,40	1,18	5		4,49	0,88	6		5,42	0,70	6		3,14	1,60	3	
0	RDW	14,54	0,47	5		14,86	0,69	5		14,86	1,08	5		14,73	0,64	3	
6		18,37	2,76	3		19,60	0,56	6		19,72	1,12	5		14,30	0,17	3	*** &&& ###
12		16,32	0,34	5		15,60	0,67	6		16,65	0,67	6		25,80	1,22	3	*** &&& ###
0	NEU	16,44	11,08	5		16,30	6,73	5		15,40	3,56	5		17,70	3,40	3	
6		24,53	4,07	3		18,47	5,54	6		15,12	4,09	5		23,80	1,95	3	
12		10,52	1,76	5		20,10	9,79	6		14,85	4,36	6		62,47	16,28	3	*** &&& ###
0	LIM	73,57	9,32	5		77,54	7,43	5		77,32	5,07	5		70,73	1,36	3	
6		64,07	6,23	3		73,73	6,34	6		78,27	3,91	5		65,03	4,61	3	
12		84,96	2,43	5		83,40	20,31	6		77,07	11,40	6		32,70	15,54	3	*** &&& ###
0	MONO	1,12	0,47	5		1,12	0,15	5		1,10	0,33	5		0,97	0,31	3	
6		0,77	0,60	3		0,67	0,40	6	#	1,50	0,27	5		2,60	1,30	3	*** &&& #
12		0,68	0,25	5		1,07	0,48	6		0,87	0,28	6		0,90	0,44	3	
0	EOS	1,96	1,45	7		1,78	1,35	6		1,31	1,11	9		0,75	0,37	4	
6		5,37	3,93	3		4,08	1,29	6		2,26	0,96	5		3,50	1,21	3	
12		1,40	0,85	5		1,10	0,69	6		4,90	9,57	6		0,60	0,62	3	
0	BASO	1,23	1,20	7		0,95	0,57	6		0,72	0,67	9		0,10	0,00	4	
6		2,40	1,44	3		1,63	0,71	6		0,92	0,16	5	*	2,30	1,04	3	#
12		1,16	0,25	5		1,20	0,52	6		0,65	0,20	6		0,97	0,70	3	
0	LUC	2,50	1,01	5		1,86	0,36	5		3,00	1,44	5		3,07	1,36	3	
6		2,83	1,44	3		1,42	0,32	6		1,94	0,40	5		2,77	0,31	3	
12		1,24	0,49	5		1,55	1,09	6		1,63	0,44	6		2,37	1,77	3	
0	PLT	1437	221	5		1641	182	5		1394	177	5		1309	658	3	
6		1990	732	3		1718	571	6		1316	212	5		1360	55	3	
12		1149	241	5		1319	218	6		1135	316	6		2920	1074	3	*** &&& ###
0	Large PLT	16,20	9,68	5		25,80	8,81	5		23,80	3,11	5		18,67	17,67	3	
6		17,00	6,24	3		19,00	12,21	6		20,60	11,39	5		3,33	0,58	3	
12		40,20	4,15	5		44,00	17,52	6		40,17	7,73	6		63,67	7,37	3	* #
